# Insufficient OPC migration into demyelinated lesions is a cause of poor remyelination in MS and mouse models

**DOI:** 10.1007/s00401-013-1112-y

**Published:** 2013-04-18

**Authors:** Amanda Boyd, Hui Zhang, Anna Williams

**Affiliations:** MS Centre, MRC Centre for Regenerative Medicine, The University of Edinburgh, Edinburgh Bioquarter, 5 Little France Drive, Edinburgh, EH16 4UU UK

**Keywords:** Remyelination, Semaphorin, Migration, Oligodendrocyte precursor cell

## Abstract

Failure of remyelination of multiple sclerosis (MS) lesions contributes to neurodegeneration that correlates with chronic disability in patients. Currently, there are no available treatments to reduce neurodegeneration, but one therapeutic approach to fill this unmet need is to promote remyelination. As many demyelinated MS lesions contain plentiful oligodendrocyte precursor cells (OPCs), but no mature myelinating oligodendrocytes, research has previously concentrated on promoting OPC maturation. However, some MS lesions contain few OPCs, and therefore, remyelination failure may also be secondary to OPC recruitment failure. Here, in a series of MS samples, we determined how many lesions contained few OPCs, and correlated this to pathological subtype and expression of the chemotactic molecules Semaphorin (Sema) 3A and 3F. 37 % of MS lesions contained low numbers of OPCs, and these were mostly chronic active lesions, in which cells expressed Sema3A (chemorepellent). To test the hypothesis that differential Sema3 expression in demyelinated lesions alters OPC recruitment and the efficiency of subsequent remyelination, we used a focal myelinotoxic mouse model of demyelination. Adding recombinant (r)Sema3A (chemorepellent) to demyelinated lesions reduced OPC recruitment and remyelination, whereas the addition of rSema3F (chemoattractant), or use of transgenic mice with reduced Sema3A expression increased OPC recruitment and remyelination. We conclude that some MS lesions fail to remyelinate secondary to reduced OPC recruitment, and that chemotactic molecules are involved in the mechanism, providing a new group of drug targets to improve remyelination, with a specific target in the Sema3A receptor neuropilin-1.

**Electronic supplementary material:**

The online version of this article (doi:10.1007/s00401-013-1112-y) contains supplementary material, which is available to authorized users.

## Introduction

The quest for discovery of molecules that improve CNS remyelination is prompted by the premise that this will not only help repair demyelinated lesions in multiple sclerosis (MS), aiding symptoms by overcoming conduction block and temporal dispersion of action potentials by restoring saltatory conduction, but also protect axons from neurodegeneration which causes progressive disability [8, 24]. Myelinating oligodendrocytes have been shown to be trophic for axons, providing them with necessary metabolic support [14, 25]. Remyelination does occur in MS brain, but is inefficient and inadequate [34–36, 38, 40]. For remyelination to occur, oligodendrocyte precursor cells (OPCs) must survive, proliferate, migrate to the lesion and differentiate into mature oligodendrocytes forming compact myelin sheaths [12]. Failure of remyelination may occur at any step in this process, but it is thought to be mostly due to failure or arrest of oligodendroglial differentiation. This is partly due to pathological studies revealing that 60–70 % of demyelinated MS lesions contain oligodendroglial cells in an arrested maturation state [6, 27] and is reflected in the molecules now associated with altering CNS remyelination in rodents, which all promote oligodendroglial maturation: 9-cis retinoic acid [17], anti-Lingo-1 antibodies [30], Wnt inhibition [10], axin-2 stabilization [11], loss of cdk2 [2], noggin [42], antibodies antagonizing death receptor-6 [29] and CXCR4 [3]. The effect of these molecules in humans is not known and only anti-Lingo antibodies have reached clinical trial in MS (Clinicaltrials.gov identifier NCT01244139).

Molecules affecting other processes necessary for remyelination, such as migration, have been less studied in spite of a third of demyelinated MS plaques containing few OPCs [6, 27]. Group 3 semaphorins are secreted factors that attach to extracellular matrix forming gradients for cells to use as migration cues. Sema3A binds to the receptor Neuropilin (NP)1 and Sema3F to the receptor NP2, and in development, Sema3A is an inhibitory and Sema3F an attractive migratory signal for OPCs [43, 45]. We have previously shown that in adulthood, Sema3A and 3F mRNA expression is absent in white matter, but re-expressed in MS lesions in a differential way in different lesions, with active lesions (more inflammatory and more likely to remyelinate) containing higher mRNA expression of the chemoattractant Sema3F than Sema3A, and chronic active lesions (less inflammatory and less likely to remyelinate) with higher mRNA expression of the chemorepellent Sema3A than Sema3F [54]. Here, we assessed the number of OPCs in our series of MS lesions in *postmortem* tissue, and correlated these with pathological classification and Sema3A/Sema3F protein expression. We found a correlation between a lower number of OPCs, chronic active lesion type and a higher expression of the chemorepellent protein Sema3A. In contrast, a low expression of the chemorepellent Sema3A and higher expression of the chemoattractant Sema3F correlates with active lesions and more variable, but generally higher OPC numbers. We then tested the hypothesis that the mechanism for these observations is due to the effect of these chemotactic factors on OPC migration and subsequent remyelination by manipulating levels of Sema3A or 3F in a mouse model of demyelination. We conclude that migration failure is an important cause of remyelination failure and introduce the Sema3A/NP1 pathway as a possible therapeutic target to improve OPC migration and remyelination in MS.

## Materials and methods

Animal work was carried out in accordance with the University of Edinburgh regulations under Home Office rules, with local ethics committee consent.

### MS brain samples

*Postmortem* unfixed frozen tissue was obtained from the UK Multiple Sclerosis Tissue Bank via a UK prospective donor scheme with full ethical approval (MREC/02/2/39). Two independent researchers characterized the lesion types as active, chronic active, chronic inactive or remyelinating [48]. Active plaques have indistinct borders on Luxol fast blue (LFB) staining, and inflammatory cells throughout the lesion (mostly large, round, and lipid-laden macrophages/microglia). Chronic active lesions have a demyelinated core with few inflammatory cells, but a ring of lipid-laden macrophages/microglia at their edge. Chronic inactive lesions have a sharp edge on LFB and few inflammatory cells throughout. We analyzed active (*n* = 9), chronic active (*n* = 6), chronic inactive (*n* = 8) and remyelinated (shadow) (*n* = 7) MS plaques from 15 blocks of brain tissue from 10 MS patients and 5 blocks from 5 controls with no neurological disease (Table 1).

**Table 1:** Characteristics of MS brain blocks analyzed

Patient	Sex	Age (years)	MS type	Disease duration (years)	Time to postmortem (h)	No. of MS lesions	Active	Chronic active	Chronic inactive
MS100	M	46	SP	8	7	11	1	0	7
MS121	F	49	SP	14	24	3	2	1	0
MS136	M	40	SP	9	10	9	1	0	3
MS147	F	60	SP	21	27	3	1	1	1
MS154	F	34	SP	21	12	4	2	0	1
MS176	M	37	PP	27	12	7	0	0	2
MS187	F	57	SP	27	13	4	0	0	0
MS207	F	46	SP	25	10	8	0	3	3
MS230	F	42	SP	19	31	4	2	0	0
MS242	F	57	SP	19	12	6	0	3	3
Control									
CO11	M	77			26				
CO14	M	64			18				
CO25	M	35			22				
CO28	F	60			13				
CO39	M	82			21				

### Immunohistochemistry/fluorescence

Colorimetric labelling (EnVision+, Dako) was used to see patterns of staining and immunofluorescence labelling to identify the cells expressing each molecule of interest, in regions of interest. Human *postmortem* tissue is much more difficult to use than mouse perfused tissue/cultures and required antigen retrieval by microwaving in Vector H-3300 antigen unmasking solution (Vector Laboratories) for 10 min, and for immunofluorescence, autofluorescence was partially suppressed using 0.01 % sudan black in 70 % ethanol for 10 min. OPCs in human tissue were identified either using Nkx2.2 antibodies for tissue processed for immunofluorescence (rabbit, 1 in 200, Developmental Studies Hybridoma Bank, University of Iowa, USA), or using a combination of being positive for Olig2 expression (goat, 1 in 100, R and D systems, or rabbit 1 in 100, Sigma) and negative for NogoA expression (Mouse 1 in 100, R and D systems), for colorimetric staining. Antibodies to NG2 or PDGFRα do not work reliably in our hands in human frozen tissue. Other antibodies: CD68 (rat, 1 in 200, Abcam), cleaved caspase-3 (rabbit, 1 in 300, Cell Signaling), GFAP (glial fibrillary acidic protein, chick, 1 in 500, Covance), Ki67 (rabbit, 1 in 400, Novocastra), myelin basic protein (MBP, rat, 1 in 300, Serotec), neurofilament (NF–H, chick, 1 in 50,000, Covance), Neuronin N (NeuN, mouse, 1 in 300, Millipore), Neuropilin-1 (Rabbit, 1 in 50, CST), Neuropilin-2 (Goat, 1 in 50, R and D systems), NG2 (mouse, or rabbit, 1 in 200, Millipore), Sema3A (rabbit, 1 in 100, Abcam), Sema3F (rabbit, 1 in 50, Millipore). Appropriate fluorescent secondary antibodies were used (AlexaFluor, Invitrogen).

For quantification of numbers of OPCs in MS lesions, consecutive 8-μm cryostat sections of MS tissue or tissue from normal controls were stained with Olig2 and NogoA antibodies and numbers of single and double-positive cells in each field of view calculated. At least three fields of view (each sized 0.2 mm^2^) were measured for each lesion, increasing up to 11 fields of view for the largest lesions. The normal range of OPC number was defined as the mean ± one standard deviation of OPC counts from 35 fields of view of white matter from blocks from five different normal controls.

For measurement of numbers of nkx2.2+, CC1+, Ki67+ (proliferation) or cleaved caspase-3+ cells (apoptosis) in cryostat sections, we counted the number of Ki67+ or cleaved caspase-3 + cells per unit area of the lesion and contralateral normal tissue in the corpus callosum on the same section, using a minimum of 20 sections from four mice per treatment group. Statistical analysis: ANOVA for multiple comparisons with Tukey’s post test and the nonparametric Mann–Whitney test for two group comparisons, with a *p* < 0.05 considered as statistically significant.

## Surgery

### Wild-type mice

Using anaesthetized 12-week-old C57Bl/6 male mice, 2 μl of 1 % lysophosphatidyl choline/lysolecithin (LPC) was injected through a hole drilled in the skull at stereotactic coordinates 1.2 mm posterior, 0.5 mm lateral, 1.4 mm deep to the bregma over 4 min using a 30 gauge needle attached to a Hamilton syringe, driven by a KD Scientific Nano pump, which was left in situ for 4 min to reduce backflow. MR scans were performed 4 days later to select mice with demyelinating lesions of similar sizes—a minimum of five mice was chosen for each treatment and time point (48 in total). Injection of 10 μg/ml of rSema3A/rSema3F/saline (all mixed with 10 μg/ml Laminin) was similarly carried out on day 6. Mice were perfused with 4 % PFA at 2, 3 or 4 weeks. Fixed brains were bisected coronally at the injection site, with the anterior portion processed for cryosectioning, and the posterior portion for electron microscopy.

To isolate tissue for Western Blot quantification of Sema3A/Sema3F protein in demyelinating lesions, unfixed brains were removed from mice at days 3 and 7 after LPC injection, the lesion area dissected out and compared with an equivalent area of non-lesioned corpus callosum. Further time points were not included as after post-injection day 7, we could no longer determine the exact site of the lesion under a dissection microscope due to remyelination. Western Blots were carried out using Sema3A antibody 1:1000, Abcam, Sema3F antibody 1:500, Millipore and GAPDH 1:10,000, Sigma, as a loading control. For quantification, the density of bands from two blots from two experiments was compared using Image J.

### Knockdown mice

To produce transgenic mice expressing reduced levels of Sema3A (Sema3AKD), we crossed Sema3A floxed mice (Riken RBRC01106) with a mouse expressing Cre recombinase under the HPRT promoter (from L. Smith, Edinburgh University, Edinburgh) (both on a CD1 background), and confirmed the genotype by PCR (http://www2.brc.riken.jp/animal/pdf/01106_PCR.pdf). Knockdown of Sema3A protein was confirmed using Western Blot (Sema3A antibody 1:1000, Abcam, GAPDH 1:10,000, Sigma, as a loading control). For quantification, the density of bands from three blots was compared using Image J. 12-week-old male Sema3AKD mice and their littermates not expressing Cre (as controls), were used for surgery as above, with five mice for each group and time point.

### Magnetic resonance imaging

MRI data were collected on anaesthetized, monitored mice using an Agilent 7T preclinical scanner (Agilent Technologies), with a 72 mm volume coil and a phased array mouse brain coil (Rapid Biomedical). Twenty-nine contiguous coronal T2-weighted fast-spin echo images (echo train length 8) of 0.4-mm slice thickness were collected with the following parameters: repetition time (TR) = 3,000 ms; effective echo time = 36 ms; field of view = 19.5 mm × 19.5 mm; matrix = 192 × 192; 6 signal averages; total scan time 7 min 18 s.

### Semi-thin sections and transmission electron microscopy (EM)

1-mm thick coronal section samples were post-fixed in 4 % PFA containing 0.5 % glutaraldehyde for 1 h, then 2 % PFA/2 % glutaraldehyde at 4 °C overnight and processed into resin blocks using standard protocols. Sagittal 1-μm semi-thin sections were stained with toluidine blue to select suitable areas for investigation. Ultra-thin sections, 60 nm thick, were stained in uranyl acetate and lead citrate. Semi-thin sections were taken throughout the lesion (all of similar size on MR) and mounted onto one slide per animal. These slides were ranked for those with the most myelinated profiles in the lesion to those with the least by two blinded observers. No attempt was made to assign a value to the proportion of remyelination, but simply to establish how a lesion ranked relative to others, as validated and used previously [17, 41, 55]. To confirm this ranking, we examined ultra-thin sections for the percentage of myelinated fibres, and the thickness of myelin sheaths. The mean percentage of myelinated fibres was measured from 10 fields per animal, accounting for at least 500 fibres. The thickness of myelin around myelinated fibres was expressed as a g-ratio (axon perimeter divided by myelinated fibre perimeter) to accommodate that myelin is thicker around larger axons than smaller axons. This was measured using Image J by tracing around a minimum of 100 individual fibres per lesion, including all myelinated fibres in each field, to avoid bias, using a pen and electronic pad (Bamboo pad, Wacom). Statistical analysis: ANOVA and post test Tukey’s multiple comparison test, with a *p* < 0.05 considered as statistically significant.

## Results

### OPC number in MS lesions

**Fig. 1:**
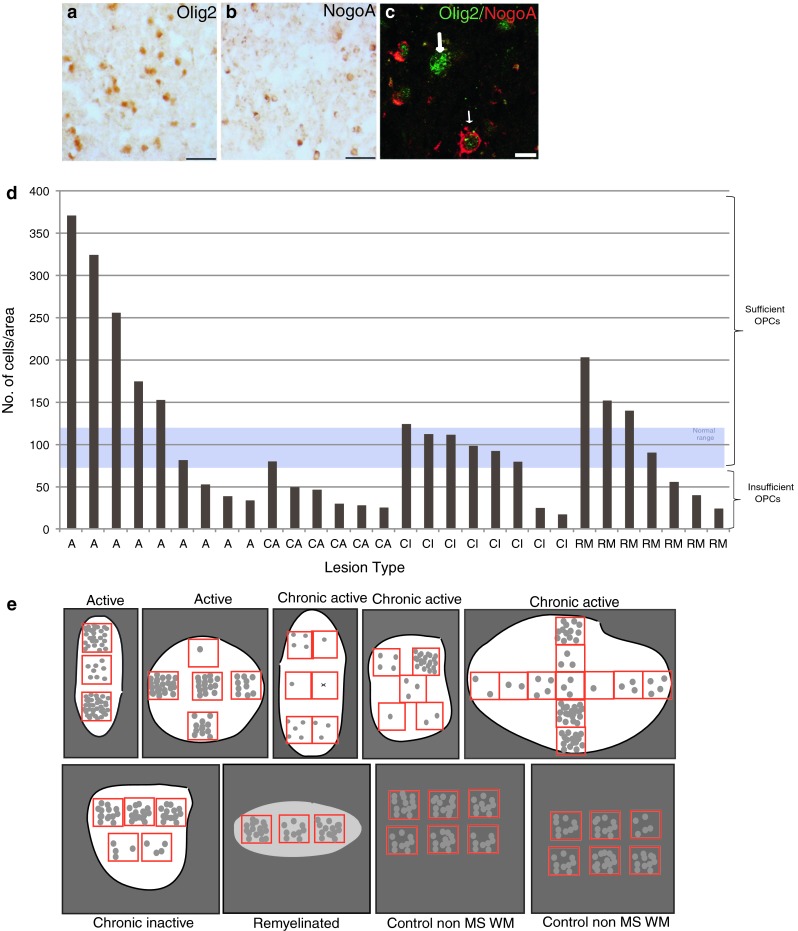
Different human MS lesions contain different numbers of OPCs. Colorimetric staining of an active MS lesion showing **a** Olig2+ cells, **b** NogoA+ cells and **c** immunofluorescence showing dual labelling (Olig2 in *green* and NogoA in *red*). The *thick arrow* shows an Olig2+ cell and the *thin arrow* a dual labelled cell. *Scale bar* **a** and **b** 40 μm **c** 10 μm. **d** *Bar* graph of average OPC number per lesion area, with one *bar* per lesion, showing the variation between pathological subtypes, with fewer OPCs in the chronic active lesions. The normal range of numbers of OPC in the brain was defined as the mean ± one standard deviation of OPC counts from 35 fields of view from 5 blocks from 5 different *postmortem* brains where death was due to a non-neurological cause. **e** Schematic showing the variability of OPC number in different regions of single lesions represented as *white* (for demyelinated lesions) or *pale grey* areas (for remyelinated lesions) compared with *white* matter of control non-MS brain tissue. *Red boxes* represent fields of view counted, and 1 *grey circle* represents 10 OPCs

Many papers state that the cause of remyelination failure in MS is due to arrest of oligodendroglial maturation within a demyelinated plaque, as most MS lesions contain sufficient OPCs. Previously, the largest series of counts of OPCs in lesions suggested that around 70 % of plaques had sufficient or plentiful OPCs, and 30 % few OPCs [27]. The best way to identify an OPC in human *postmortem* tissue has been debated, mostly due to the unreliability of staining using antibodies to NG2, PDGFRα and Nkx2.2 in this tissue, which is difficult to use. However, as used in [21], a combination of positive staining with Olig2 (a nuclear marker which stains OPCs and more mature oligodendrocytes) and a lack of staining with NogoA (cytoplasmic staining in mature oligodendrocytes) provides reliable staining throughout MS tissue (Fig. 1a–c).

We used this method to assess the number of OPCs throughout lesions from *postmortem* MS tissue obtained from the UK MS Tissue Bank. We analyzed active (*n* = 9), chronic active (*n* = 6), chronic inactive (*n* = 8) and remyelinated (shadow) (*n* = 7) MS plaques from 15 blocks of brain tissue from 10 MS patients and five blocks from five controls (Table 1). Active plaques are thought to have more propensity to remyelinate as compared to chronic plaques and chronic active plaques occupy the middle zone. The normal range of numbers of OPC in the brain was defined as the mean ± one standard deviation of OPC counts from 35 fields of view from five blocks from five different *postmortem* brains, where death was due to a non-neurological cause (mean 95 ± 23 SD).

We found that in MS lesions, the average OPC count (Olig2+/NogoA−) per lesion for active lesions was higher than normal in 5/9 lesions examined, with one within the normal range and three with low counts. In remyelinated lesions, OPC number was higher than normal in 3/7 lesions, with one within the normal range and three with low counts. For chronic inactive lesions, one lesion had a count just higher than normal, 5/6 lesions had a normal OPC count, with two with a low count. However, 5/6 chronic active lesions had lower than normal counts, with one just reaching the normal range. Therefore, in total, 11/30 (37 %) of MS lesions in our sample had insufficient OPCs, and although this occurred in every lesion type, most chronic active lesions contained few OPCs (Fig. 1d). This confirms the previous results [27], and rebuts the idea that OPC maturation failure is the only important cause of remyelination failure in MS. Furthermore, we noted that the density of OPCs in lesions was variable within different areas of the lesion (Fig. 1e), which is rarely considered although it has been described previously [5]. This was true of all types of lesions, and we found no correlation of local number of OPCs and position within the lesions (e.g., centre or edge) nor with the density of microglia/macrophage infiltrate. In comparison, OPC densities appeared less variable in white matter in control non-MS brains.

### Sema3A and Sema3F are differentially expressed in MS lesions

The existence of lesions containing few OPCs, and the predominance of chronic active lesions in this group, which are thought to be less likely to remyelinate, raised the hypothesis that these lesions may be either expressing a chemorepellent for OPCs, or failing to express a chemoattractant, thus reducing OPC recruitment. Our previous work showed that the chemotactic factors Sema3A and 3F are re-expressed in MS lesions at the mRNA level, as compared to control white matter [54]. However, the protein expression of Sema3A or 3F has not previously been described in adult human brain in either normality or pathology. We used colorimetric staining to identify patterns of Sema3A/3F expression in lesions, and immunofluorescence to focus in on which cells expressed these molecules.

**Fig. 2:**
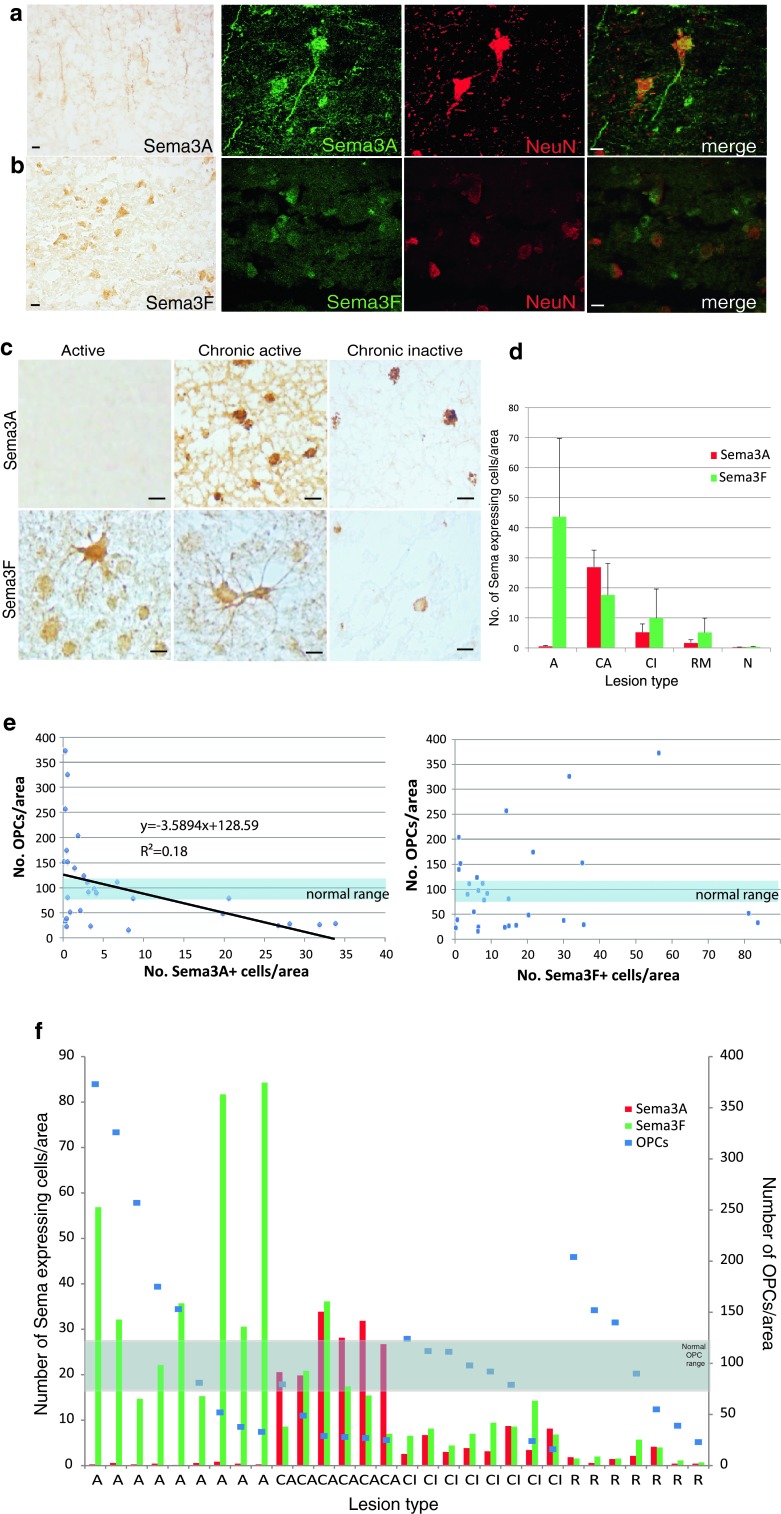
MS lesions with different pathology show different patterns of protein expression of Sema3A and 3F. In normal human brain *grey* matter, there is expression of Sema3A protein in neuronal cell bodies and some axons (**a**) and Sema3F (**b**) in neuronal cell bodies by colorimetric and immunofluorescence staining (*green* Sema3A/F labelling, *red* NeuN staining neuronal cell bodies). **c** Colorimetric staining shows patterns of staining in active, chronic active or chronic inactive MS lesions. There is absence of Sema3A staining, but florid Sema3F staining in active lesions, as compared to more chronic lesions, which express both proteins. *Scale bars* 10 μm. **d** Quantification of the number of cells in each type of MS lesion expressing Sema3A or Sema3F/unit area confirms that there are few cells in active lesions expressing Sema3A, but more expressing Sema3F, whereas in chronic active lesions, more cells express Sema3A compared with Sema3F, and chronic inactive and remyelinated lesions have few cells expressing either protein. **e** For each lesion, the number of cells expressing Sema3A or Sema3F is plotted against the average number of OPCs in the lesion, to identify a correlation. The normal range of number of OPCs is shown as defined as the mean ± one standard deviation of OPC counts from 35 fields of view from 5 blocks from 5 different *postmortem* brains where death was due to a non-neurological cause. An increase in number of cells expressing Sema3A correlates with fewer OPCs found in the lesion, whereas the relationship is less clear with Sema3F. **f** Graph showing numbers of cells expressing Sema3A or Sema3F and the average number of OPCs in each lesion, separated by pathological subtype. The normal range of OPC number is marked, as described above. Although there is variability between lesions, if the lesion contains more Sema3A-expressing cells (*red*), the OPC number is lower, and high numbers of OPCs are only present if the number of Sema3A-expressing cells is low

**Fig. 3:**
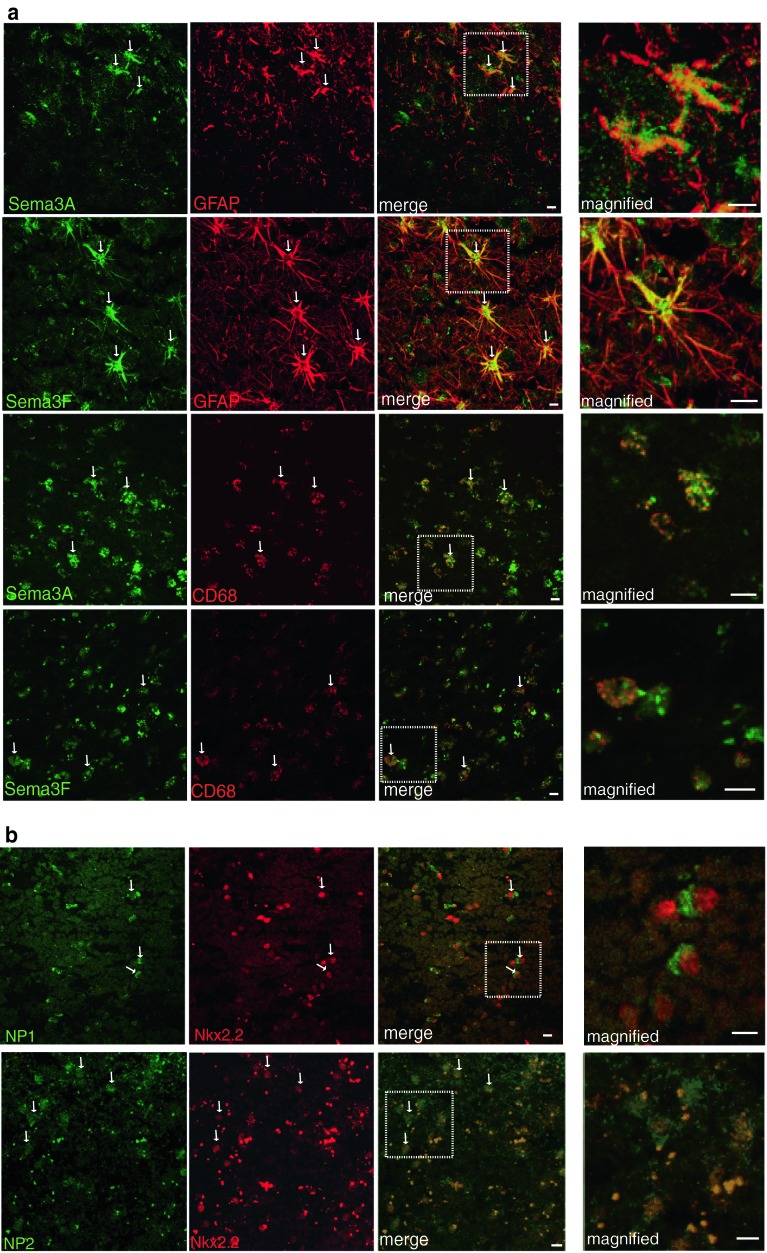
Astrocytes and microglia/macrophages express Sema3A, 3F in MS brain, and oligodendroglial cells express their receptors **a** Astrocytes (GFAP-labelled) and microglia/macrophages (CD68-labelled) in chronic active lesions express Sema3A. Astrocytes and microglia/macrophages in active and chronic active lesions express Sema3F (active lesion shown) **b** NP1 and NP2 are expressed in oligodendroglial cells (Nkx2.2-labelled) (chronic active lesions shown). Examples of double-positive cells are labelled with *arrows*. Pictures are flattened confocal stacks. *White boxes* indicate location of magnified picture. High background staining is found in this human *postmortem* tissue and is most obvious in the NP2 photographs where the bright punctate staining is nonspecific. *Scale bar* 10 μm

Normal control brain tissue showed expression of Sema3A and 3F protein in neuronal cell bodies plus Sema3A in some axons in the grey matter (Fig. 2a, b), but no detectable expression in the white matter. In active plaques, Sema3F was present in and around the lesion, labelling both astrocytes and microglia/macrophages, but virtually no Sema3A protein expression was seen, despite the positive staining on the same sections in neurons in the grey matter (Fig. 2a), with only an occasional perivascular Sema3A-positive cell seen. However, in chronic active lesions, there was both Sema3F and 3A staining (again in astrocytes and microglia/macrophages) with more Sema3A present at the active rim. Chronic inactive MS plaques contained a few Sema3A or 3F-positive microglia/macrophages only. Remyelinated plaques contained very few Sema3A or Sema3F-positive microglia/macrophages only (Figs. 2c, d, 3a).

Thus, we have found a correlation between the activity of a MS lesion, defined pathologically, and the presence of expression of the chemorepellent Sema3A. We found that the number of OPCs present in any lesion inversely correlated with the density of Sema3A-positive cells in that lesion, but that the pattern was less clear for Sema3F expression and OPC number (Fig. 2e). As we showed in Fig. 1d that OPC numbers are variable between lesions even of the same pathological subtype, we compared the number of cells expressing Sema3A and 3F in each lesion grouped by subtype, with the number of OPCs found in that lesion (Fig. 2f). Although there is individual variation, there is again a pattern that the presence of Sema3A-positive cells in the lesion correlates with low OPC numbers, and that these are mostly chronic active lesions. There is less correlation between the number of cells expressing the chemoattractant Sema3F and the number of OPCs, although active lesions generally contain less Sema3A+ cells, more Sema3F+ cells and more OPCs.

Therefore, the presence of the chemorepellent Sema3A correlates with fewer OPCs in the lesion, and a chronic active pathological subtype, which has a lower propensity to remyelinate.

### OPCs express the receptors for Sema3A and 3F

Sema3A and 3F bind to the transmembrane receptors Neuropilin (NP) one or two respectively. These receptors are distinct though chemically related, with no crossover in binding of the Sema3A or 3F ligands [15]. OPCs in human MS tissue express the neuropilin receptors (Fig. 3b), although these receptors are also expressed by microglia/macrophages and astrocytes in all MS lesion types (Suppl. Fig. 1) except remyelinated lesions (data not shown).

### Sema3A and 3F expression are regulated in demyelinating lesions in mouse

These human data suggest that failure of OPC recruitment is important in a subset of MS lesions, and that the mechanism of this may involve Sema3A and 3F. However, research on human *postmortem* MS tissue is by its nature confined to one time point, so we turned to a model of demyelination in the mouse to investigate whether this correlation was causal.

**Fig. 4:**
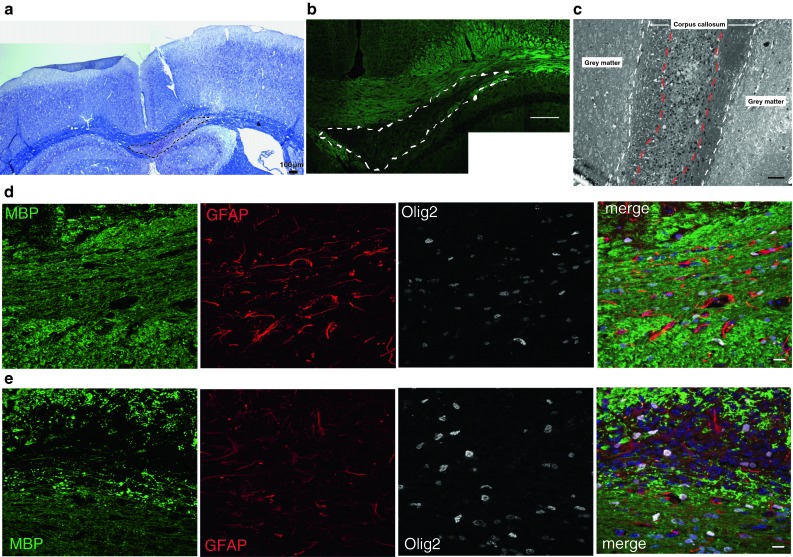
Demyelination in our mouse model **a** typical demyelinated lesion shown by Luxol fast blue staining: demarcated by *dashed line*. *Scale bar* 100 μm. **b** Typical lesion shown by fluoromyelin *green* staining (composite photo)—demarcated by *dashed line*. *Scale bar* 200 μm. **c** Semi-thin section stained with toluidine *blue* showing a lesion, delineated with *red dashed line*, in the corpus callosum delineated with *white dashed line*. *Scale bar* 100 μm. Immunofluorescence staining of normal side (**d**) and lesioned side **e** of corpus callosum with antibodies against MBP (*green*), Olig2 (*white*), GFAP (*red*) and a merge with Hoechst to stain nuclei (*blue*). *Scale bar* 10 μm

**Fig. 5:**
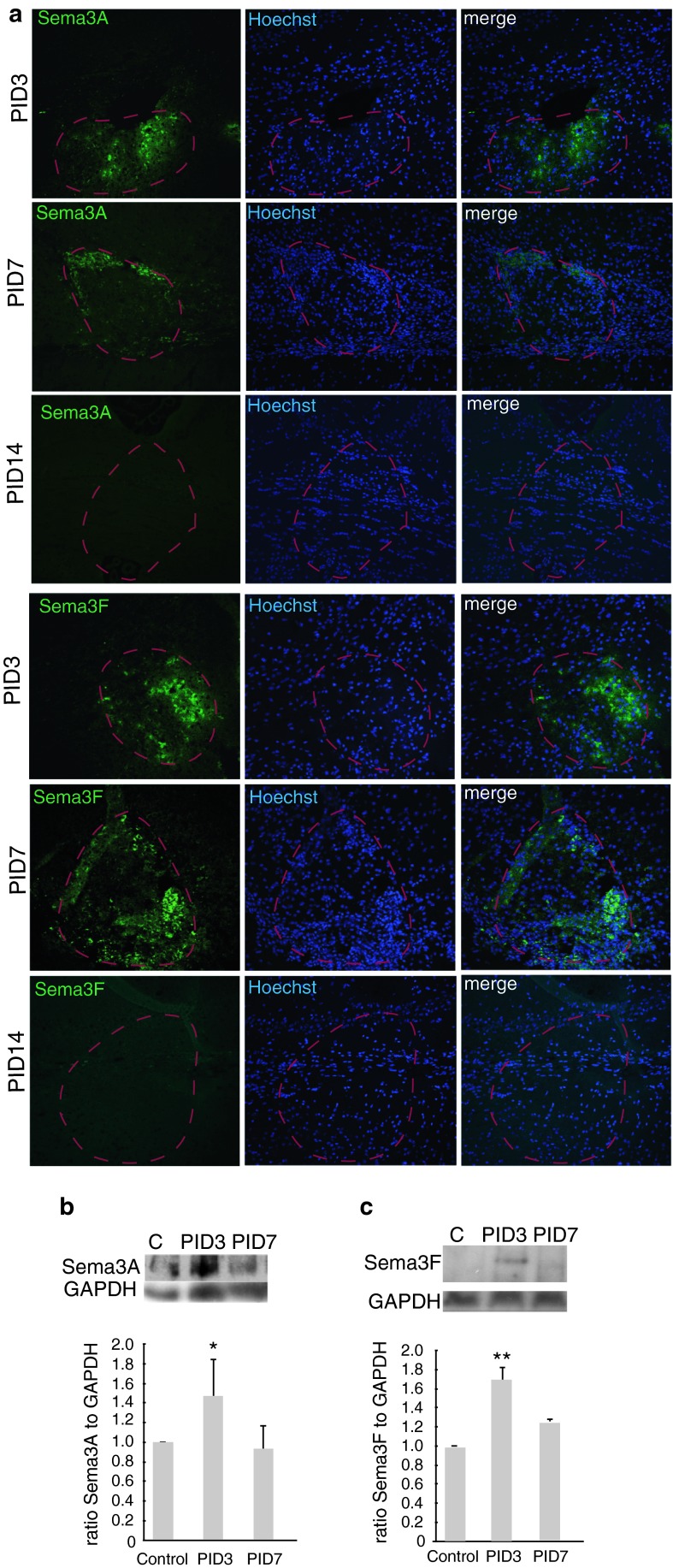
Sema3A and 3F expression in our mouse model of demyelination. **a** Immunofluorescence for Sema3A or Sema3F in a mouse lesion at post-injection days (PID) 3, 7 and 14, with Sema3A or Sema3F (*green*), Hoechst to stain cell nuclei (*blue*) and a merge. The lesion is delineated by a *red line*. *Scale bar* 100 μm. Western blot analysis confirms this transient rise in expression for Sema3A (**b**) and Sema3F (**c**). *C* control unlesioned corpus callosum, PID 3,7 shown. GAPDH is loading control. Densitometry blots from 2 separate sets of animals for each, showing mean density ratio plus SD of Sema3A/F band to GAPDH band, with ratio in unlesioned tissue defined as 1 (*p < 0.05, **p < 0.01, *t* test, when compared with control)

**Fig. 6:**
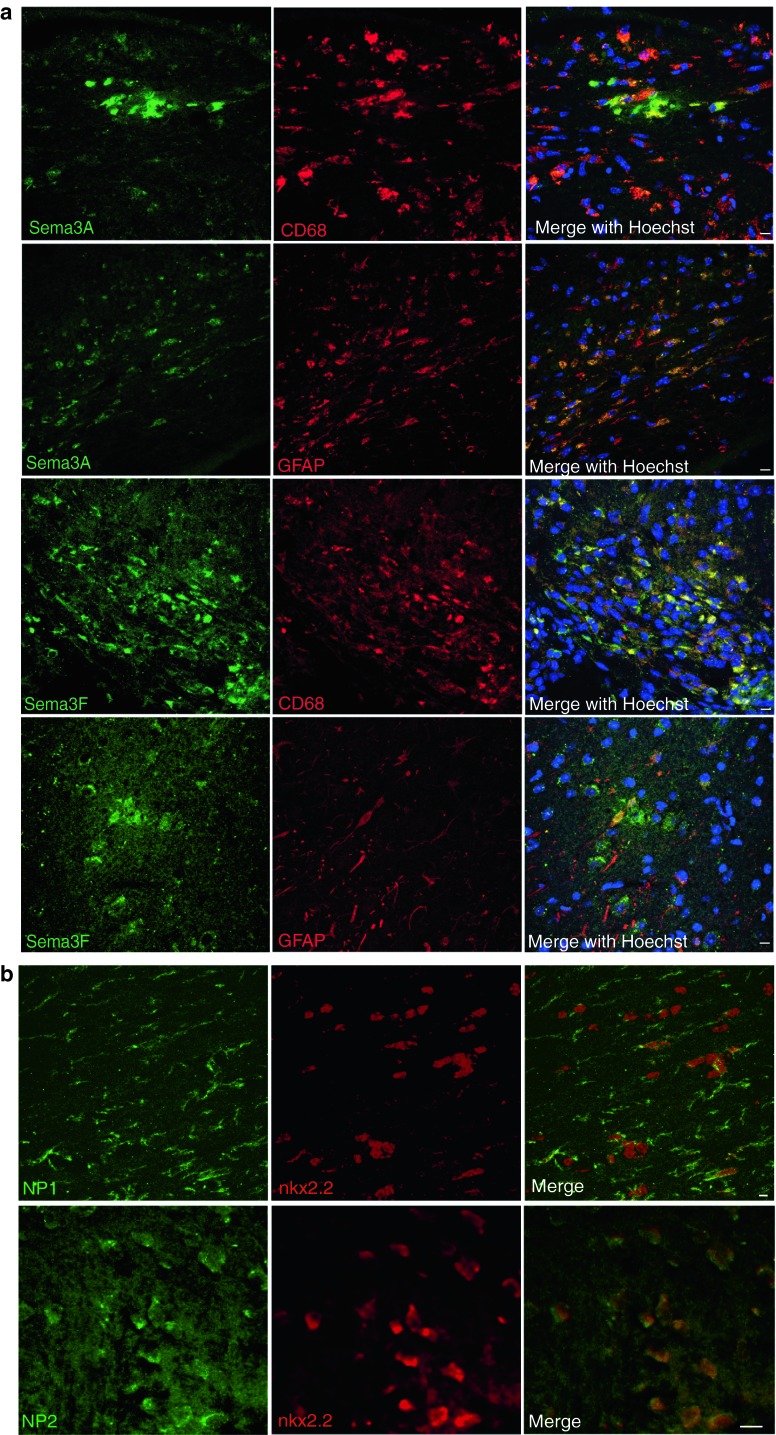
Astrocytes and microglia/macrophages express Sema3A, 3F in demyelinated lesions in our mouse model, and oligodendroglial cells express their receptors **a** Astrocytes (GFAP-labelled) and microglia/macrophages (CD68-labelled) in demyelinated lesions at post-injection day 3 express Sema3A. **b** NP1 and NP2 are expressed in oligodendroglial cells (nkx2.2-labelled) at post-injection day 3. Pictures are flattened confocal stacks. *Scale bar* 10 μm

First, we investigated whether Sema3A and 3F were expressed in a similar manner in demyelinated lesions in mouse. We created demyelinated lesions in the right side of the mouse corpus callosum by stereotactic injection of LPC (Fig. 4) and confirmed the presence of an appropriately sized and placed lesion by MR scanning. In this model, demyelination is complete around 3 days, migration of OPCs into the lesion starts at around day 6, the differentiation phase occurs around days 10–14 and remyelination is usually complete by about 4 weeks [17, 55], as remyelination failure does not occur. Sema3A and 3F are not expressed at the protein or mRNA level by immunofluorescence or in situ hybridization [54] in normal white matter in adult mice. Sema3A protein is detectable throughout demyelinated lesions at 3 days after LPC injection (PID3), but is reduced and limited to the lesion edge after 7 days (PID7). Sema3F protein expression is patchy within the lesion at day 3 (PID3), declining in a similar, but slower manner from the inner core of the lesion outwards, leaving a positive rim. By day 14 (PID14), both Sema3A and 3F staining is absent (Fig. 5a). We confirmed this transient increase in expression of Sema3A by Western Blot analysis (Fig. 5b, c). As in human MS lesions, we found that the cells expressing Sema3A or 3F in these lesions were astrocytes and microglia/macrophage and that some OPCs expressed NP1 and NP2 (Fig. 6).

### Adding rSema3A or 3F to demyelinated lesions in mouse alters OPC recruitment

As semaphorins are re-expressed after demyelination in mouse, similarly to in human MS lesions, we hypothesized that manipulation of the levels of Sema3A or 3F to a demyelinated lesion would alter OPC recruitment and hence remyelination.

**Fig. 7:**
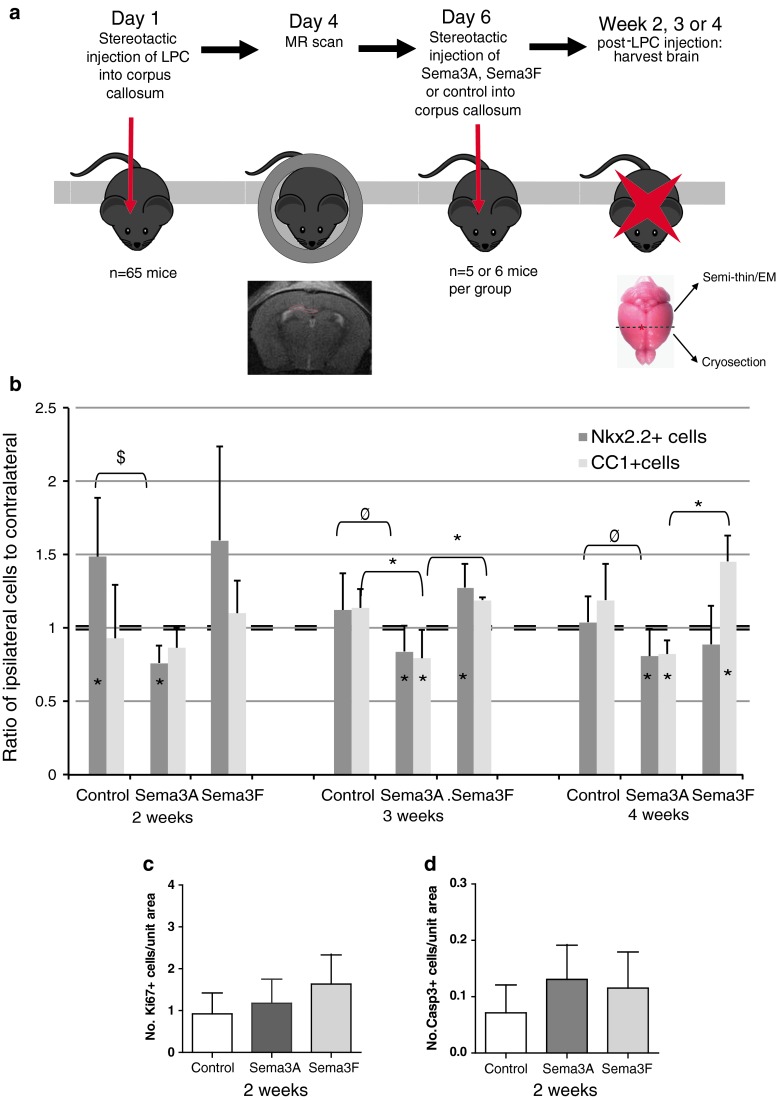
Manipulation of Sema3A or Sema3F levels in the mouse model in vivo changes OPC migration to the lesion. **a** Timeline for experiment. **b** More nkx2.2+ oligodendroglial cells are present around and within lesions treated with rSema3F and less around lesions treated with rSema3A as compared to the unlesioned sides at 2 and 3 weeks after the lesion. More CC1+ oligodendrocytes are present around and within lesions treated with rSema3F and less around lesions treated with rSema3A as compared to the unlesioned side at 3 and 4 weeks after the lesion (mean ratio + SD *asterisks* on *bars* indicate value is significantly different as compared to contralateral side (assigned value of 1), *symbols* above *bars* indicate significant differences between groups *p < 0.05, ^$^ *p* < 0.001, ^φ^ *p* < 0.01, comparing treatments by ANOVA and Tukey’s post test). There is no difference in proliferation (Ki67+ cells) **c** or apoptosis (cleaved caspase-3+ cells) **d** within the lesion between the groups at 2 weeks (mean + SD, *n* > 20 sections from 5 mice per group)

Six days after lesion induction, when demyelination is complete and migration of cells into the lesion starts [55], we injected recombinant (r)Sema3A, rSema3F or saline into the lesion mixed with laminin, to which Sema3A and 3F bind [7, 9], to aid maintenance of a high concentration local to the lesion. We then examined the lesions at 2, 3 and 4 weeks (Fig. 7a).

At 2 weeks, both control and rSema3F-injected mice showed an increased number of nkx2.2+ OPCs around the lesion as compared to the non-lesion side, presumably due to up-regulation of endogenous pro-migration molecules locally in control lesions. However, at this time point, rSema3A treated mice had significantly fewer OPCs on the lesion side as compared to the opposite side, indicating a repulsive effect that overcomes even these endogenous pro-migration factors. The effect of rSema3A is maintained even 4 weeks after the lesion with still fewer OPCs on the treated side. In animals treated with rSema3F, the increase in OPCs is maintained at 3 weeks, whereas in controls the number normalizes, but at 4 weeks, the numbers in rSema3F-treated mice are indistinguishable from controls (Fig. 7b).

The number of CC1+ mature oligodendrocytes in lesions increased in the control group and in those treated with rSema3F in line with an increase of OPC migration into these lesions and their subsequent differentiation and remyelination. However, the numbers of CC1+ cells in lesions treated with Sema3A remained significantly reduced, corresponding to reduced OPC migration and therefore less OPCs to then differentiate (Fig. 7b). If this reduced number of CC1+ cells in rSema3A-treated lesions is purely an effect of reduced differentiation of OPCs, as previously suggested [46], then we would expect a normal or increased number of OPCs in the rSema3A-treated lesions, rather than a reduction. Differences in OPC number were also not due to differences in OPC proliferation or apoptosis as there was no difference in the number of Ki67+ or cleaved caspase-3+ cells in the lesions between groups. At 2 weeks after the lesion, when most proliferation and death would be expected, there were few cells undergoing proliferation or apoptosis, and only very rarely were these found to be OPCs (Fig. 7c, d, Suppl. Fig. 2).

### Adding rSema3A and 3F to demyelinated lesions in mouse alters remyelination

**Fig. 8:**
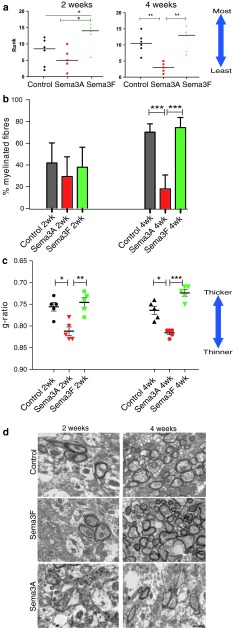
Manipulation of Sema3A or Sema3F levels in the mouse model in vivo changes remyelination efficiency. **a** Blinded ranking of remyelination shows improved remyelination with addition of rSema3F and an inhibition of remyelination with addition of rSema3A to the lesion. *Horizontal line* represents median (**p < 0.01 and *p < 0.05 using 1-way ANOVA and Tukey’s post test.) **b** Graph of percentage of myelinated fibres in each group, counted from 10 fields of view per animal, and at least 500 fibres per group. This shows that there is no difference between the number of fibres myelinated at 2 weeks, but there is a clear difference at 4 weeks, with no increase in myelinated fibres in the Sema3A-treated group over this time. In contrast, the number of myelinated fibres returns to normal levels for the corpus callosum in control and Sema3F-treated animals (mean ± SEM ***p < 0.001, **p < 0.01 and *p < 0.05 using 1-way ANOVA and Tukey’s post test). **c** Graph showing that the average g-ratio per animal in each group is significantly higher in Sema3A-treated animals at both time points, indicating thinner myelin than in the other two groups. Sema3F-treated animals have a trend to thicker myelin by 4 weeks (lower g-ratio). Note that the *y*-axis is inverted and does not start at zero, so that the graphs in **a**, **b** and **c** can be directly compared (mean ± SEM ***p < 0.001, **p < 0.01 and *p < 0.05 using 1-way ANOVA and Tukey’s post test). **d** Electron microscope photographs through lesions. *Scale bar* 1 μm

Thus, we can manipulate the number of OPCs that are recruited to mouse demyelinated lesions using rSema3A or 3F, but we next asked whether this difference in recruitment translated into a difference in remyelination efficiency. We assessed the extent of remyelination in these lesions at 2 and 4 weeks using a previously described blinded ranking system [17, 41, 55] (see methods) (Fig. 8a), by measuring the percentage of fibres which were myelinated (Fig. 8b) and the thickness of these myelinated fibres (Fig. 8c). The ranking measure showed an improvement of remyelination with Sema3F treatment and reduced remyelination with Sema3A treatment. At 2 weeks, there was no difference between the numbers of fibres myelinated between the groups, but the myelin was significantly thinner in those mice treated with the chemorepellent Sema3A (mean g-ratio 0.81 ± 0.010 (SEM), as compared to control and Sema3F (mean 0.76 ± 0.010 SEM and mean 0.75 ± 0.011 SEM, respectively). At 4 weeks, there was a clear difference in the percentage of myelinated fibres between groups, with control and Sema3F-treated mice reaching normal levels [23] (mean 70.9 % ± 2.9 SEM and mean 75.1 % ± 2.1 SEM, respectively), whereas in Sema3A-treated mice the percentage remains low (mean 18.7 % ± 4.3 SEM) and unchanged to that at 2 weeks. At 4 weeks, Sema3A-treated mouse remyelinated axons remain significantly thinner (mean g-ratio 0.82 ± 0.004 SEM) than in control (mean g-ratio 0.76 ± 0.009 SEM) and Sema3F-treated groups (mean g-ratio 0.72 ± 0.007 SEM). Although not statistically significant, Sema3F-treated mice remyelinated axons have a trend to having thicker myelin than controls at both 2 and 4 weeks, perhaps accounting for these lesions being ranked as more remyelinated at 2 weeks. The distribution of g-ratios compared to axon diameter for each group is shown in Suppl. Fig. 3.

### Improvement of OPC recruitment and remyelination in Sema3AKD mice

**Fig. 9:**
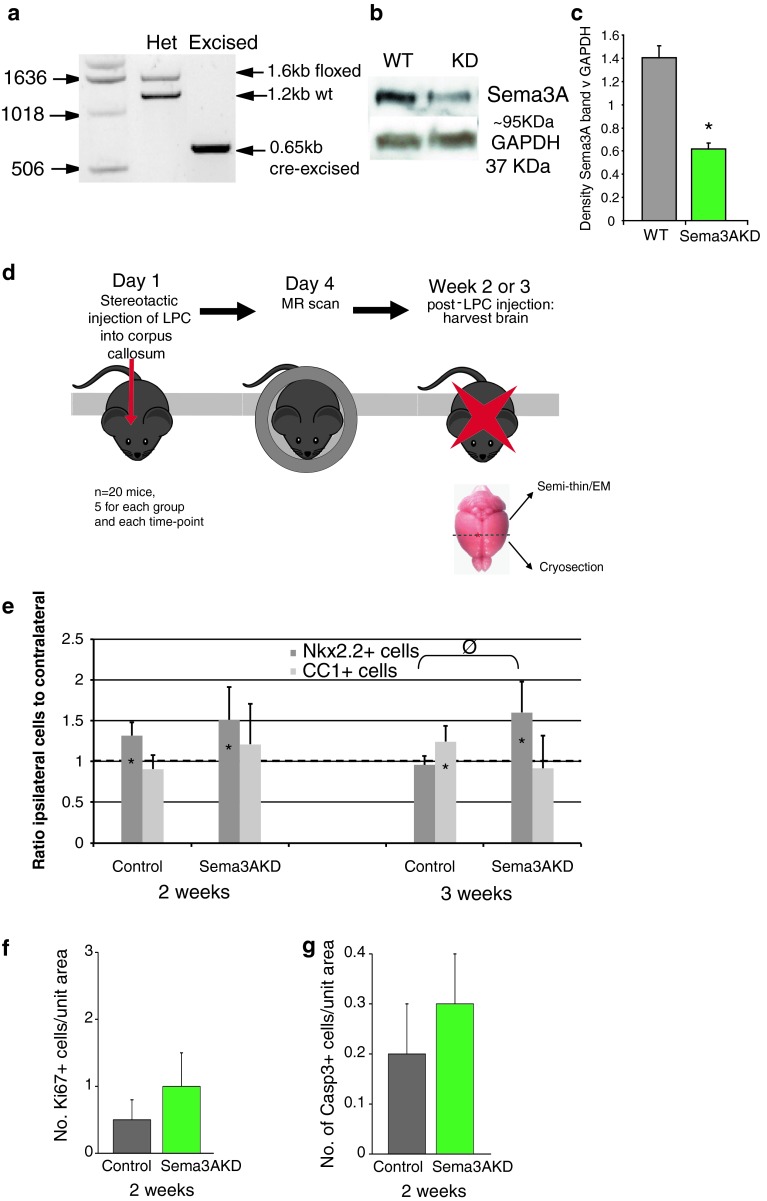
Transgenic knockdown of Sema3A increases recruitment of OPCs after demyelination in vivo. **a** PCR shows presence of Cre-excised band in Sema3AKD mice. **b** Western blot shows reduced levels of Sema3A protein in Sema3AKD mice (GAPDH as loading control). **c** Densitometry measurements of three western blots shows a significant (*p* < 0.001, *t* test) reduction in Sema3A protein expression as compared to GAPDH expression, of around 60 %. **d** Strategy for in vivo experiment. **e** More nkx2.2+ oligodendroglial cells are present around and within lesions in Sema3AKD mice as compared to the unlesioned side at 2 and 3 weeks after the lesion. CC1+ oligodendroglial cells are relatively unchanged around and within lesions in Sema3AKD mice as compared to the unlesioned side at 2 and 3 weeks after the lesion, suggesting that by these time points CC1+ number has already reached normal, correlating with remyelination of around 60 % of fibres already by 2 weeks (see Fig. 10b) (mean ratio + SD *asterisks* on *bars* indicate value is significantly different as compared to contralateral side (assigned value of 1), *symbols* above *bars* indicate significant differences between groups *p < 0.05, ^φ^ *p* < 0.01, comparing treatments by ANOVA and Tukey’s post test). **f** There is no difference in proliferation (Ki67+ cells) or apoptosis (CC1+ cells) (**g**) within the lesion between the groups at 2 weeks after injection (Mean + SD, *n* > 20 sections from 5 mice per group)

As addition of rSema3A to demyelinated lesions both reduces OPC recruitment and remyelination, we next determined whether reducing Sema3A production in lesions could improve OPC recruitment and allow efficient remyelination. We generated transgenic mice by crossing mice expressing Cre recombinase in all cells with mice containing a floxed Sema3A gene, generating Sema3A-knockdown (KD) mice (see methods) (Fig. 9a–c). Sema3A−/− mice generated using the same floxed construct show some cranial and peripheral nerve projection abnormalities [47], similarly to that found in other Sema3A−/− mice, but with no identified myelin abnormalities [1, 4]. The efficiency of Cre recombinase-mediated excision of DNA depends on the position of the loxp sites within the gene, and the distance between the sites [50], and in these transgenic mice, Sema3A protein expression in the brain was not completely knocked out, but instead reduced by around 60 % as compared to control non-Cre containing littermates (Fig. 9c).

Demyelinated lesions were made in the corpus callosum of Sema3AKD mice and their littermate controls (expressing normal levels of Sema3A) as before and lesions examined 2 and 3 weeks after, as we predicted an increase in remyelination efficiency (Fig. 9d).

**Fig. 10:**
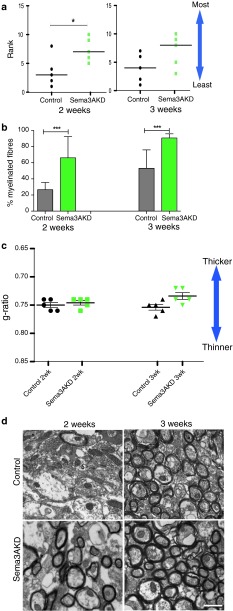
Transgenic knockdown of Sema3A in vivo changes remyelination efficiency. **a** Blinded ranking of remyelination shows more remyelination in Sema3AKD mice at 2 weeks compared to controls. *Horizontal line* represents median. *p < 0.05 using 1-way ANOVA and Tukey’s post test. **b** Graph of percentage of myelinated fibres in each group, counted from 10 fields of view per animal, and at least 500 fibres per group. This shows that there is an increase in the number of fibres myelinated at both time points in the Sema3AKD group (mean ± SEM ***p < 0.001 using 1-way ANOVA and Tukey’s post test). **c** Graph showing that the average g-ratio per animal in each group is not significantly different in Sema3AKD animals at either time point, however, Sema3AKD mice (similarly to Sema3F-treated mice, see Fig. 8c) have a trend to thicker myelin by 3 weeks (lower g-ratio). Note that the *y* axis is inverted and does not start at zero, so that the graphs in **a**, **b** and **c** can be directly compared (mean ± SEM using 1-way ANOVA and Tukey’s post test). **d** Electron microscope photographs through lesions. *Scale bar* 1 μm

We counted the number of OPCs around and within the lesions to determine whether reducing Sema3A expression affects OPC migration, and observed an increase in OPCs on the lesioned side compared to the contralateral side at 2 weeks in both control and Sema3AKD groups. However, the high number of OPCs on the lesioned side in Sema3AKD mice was maintained at 3 weeks, after OPC number normalized in the controls, similarly to rSema3F treated lesions described above (Fig. 9e). Mature oligodendrocyte numbers (CC1+) increased in the control group over time, as compared to the unlesioned side as before. However, in the Sema3AKD group, there was not much change in the number of CC1+ cells around and within lesions as compared to the unlesioned side, suggesting that by these time points the number of CC1+ cells has already reached normal, correlating with remyelination of around 60 % of fibres already by 2 weeks (Fig. 10b). There was little evidence of proliferation or apoptosis in lesions at 2 weeks after injection, and no difference between the two groups (Fig. 9f, g).

Assessment of remyelination using the same methods as before showed a clear increase in the percentage of fibres in a lesion that were remyelinated in Sema3AKD mice as compared to control mice, and no statistically significant difference in the thickness of the myelin sheaths, which remain thinner than normal in all groups. However, as in the Sema3F-treated group described before, there is a trend to increased myelin thickness in Sema3AKD mice, especially at smaller axon diameters (Fig. 10, Suppl. Fig. 4).

## Discussion

We have shown that the number of OPCs present in MS lesions in *postmortem* brain is highly variable, both within lesions and between lesions. When compared with OPC counts in normal control white matter brain tissue, on average, within a lesion, we found lower than normal numbers of OPCs in 11/30 lesions (37 %), in the normal range in 9/30 lesions (30 %), and increased numbers in 10/30 lesions (33 %). This is in line with previous work [27]. We observed that the average OPC number correlated with the pathological subtype of the lesions, with most chronic active MS lesions (thought less likely to remyelinate) containing reduced numbers of OPCs, whereas most active MS lesions (thought most likely to remyelinate) contain more OPCs as compared to normal non-diseased white matter. Many chronic inactive lesions contain sufficient OPCs, suggesting that the failure of remyelination in this lesion subtype may be due to an arrest of maturation. Most remyelinated lesions contain sufficient or increased numbers of OPCs, in line with their successful repair.

**Fig. 11:**
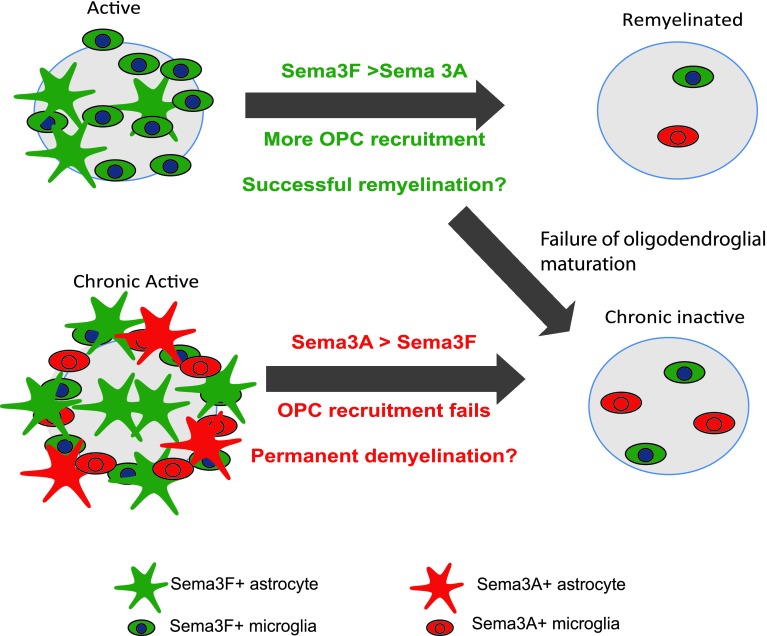
Model summarizing Sema3A and Sema3F expression in different pathological subtypes of MS lesions and correlation with success or failure of OPC recruitment and subsequent remyelination

As this suggested that different levels of OPC migration to MS lesions may influence remyelination capacity, we examined the protein expression of known chemotactic factors for OPCs—Sema3A and 3F within MS lesions. We found that although the chemoattractant Sema3F was expressed in both active and chronic active lesions, the chemorepellent Sema3A was mostly expressed in chronic active lesions, which mostly contain few OPCs. We had previously detected Sema3A mRNA in active MS lesions [54], but now describe little protein expression, and this discrepancy is not unprecedented within the brain, as PMP22 mRNA is expressed in oligodendrocytes, but no PMP22 protein is present [22]. The receptors for these ligands (NP1 and NP2) are expressed by OPCs in and around all types of MS lesions, as well as by astrocytes and microglia/macrophage, suggesting that it is the differential expression of Sema3A and 3F in different MS lesions which confers different OPC recruitment patterns and their subsequent capacity to remyelinate (Fig. 11).

We tested this hypothesis using our in vivo mouse model for demyelination and remyelination, by manipulating the levels of Sema3A or 3F using recombinant protein injected into demyelinated lesions or by using a transgenic mouse with reduced Sema3A expression. We showed that increasing Sema3F or reducing Sema3A expression increased OPC recruitment to lesions and accelerated remyelination, whereas increasing Sema3A in lesions decreased OPC recruitment and reduced remyelination, without seeing an effect on OPC proliferation, apoptosis or differentiation. This finding is discrepant with the previous finding of inhibition of rat OPC differentiation with rSema3A [46], and this may perhaps be explained by the use of different doses, at different times after demyelination and in a different species. Furthermore, if the effect here is purely due to the fact that Sema3A reduces OPC differentiation in vivo, we would expect an increase or at least maintenance of OPC number in lesions due to an arrest of their differentiation into mature oligodendrocytes, and we saw a decreased number. Thus, although we cannot exclude some effect on OPC differentiation, it is clear that this is not the key process affected in our model. One may speculate that factors promoting OPC migration will always inhibit differentiation, to avoid maturation to less motile differentiated oligodendroglial cells. However, CXCL1 has been shown to be a promoter of OPC migration, proliferation and differentiation [49, 51], and so this is clearly over-simplistic, and the effect of a cytokine may depend on timing, concentration, receptor and downstream signalling molecule expression. An alternative explanation of our findings is that OPC migration to all lesions is adequate and similar but there is differential death of OPCs in MS lesions with more death in response to Sema3A and less in response to Sema3F. Although we did not see a change in numbers of OPC undergoing apoptosis at 2 weeks after lesion between control and treated groups when there is already a difference in OPC numbers, we cannot exclude that death and clearance of OPCs may occur very fast and before this time point.

We previously showed that lentiviral delivery of Sema3F to a demyelinated lesion in a different mouse model improved remyelination [37], giving confidence in these results; an important factor considering the number of drug targets that look promising in initial publications, but which later fail to be repeated by pharmaceutical companies [39]. However, this is the first demonstration that Sema3A treatment of demyelinated lesions in the mouse brain reduces OPC migration to the lesion and inhibits its subsequent remyelination. We hypothesize that the continued presence of Sema3A expression in human MS lesions, in contrast to the expression for a short time in mouse demyelinated lesions, may be one reason why mouse demyelinated lesions remyelinate efficiently and many human MS lesions do not. This is one problem with using rodents as models for MS, as in contrast to humans, OPC recruitment and maturation normally occurs successfully in response to demyelination and remyelination does not normally fail.

Our finding that manipulation of OPC migration influences remyelination in a mouse model and the correlative human data suggests that failure of remyelination in a third of MS lesions is due to failure of OPC recruitment to these lesions. This reinstates OPC migration as an important target for therapies to improve remyelination in demyelinating disease, and introduces the Semaphorin pathway as a specific target. The most logical target is inhibition of the Sema3A pathway as this is expressed in those lesions that fail to remyelinate. The receptor for Sema3A is NP1, which is a transmembrane receptor present on OPCs, but also on macrophage/microglia [28] and T-regulatory cells (Treg) [53, 56]. We have confirmed expression of NP1 and NP2 on microglia/macrophages in culture and in vivo in mouse and human (Suppl. Fig. 1 and data not shown) raising the possibility that Sema3A is also involved in influencing migration of these cells to lesions. Thus, drug inhibition of the Sema3A pathway may increase microglia/macrophage recruitment to demyelinated lesions, which may add further benefit as microglia/macrophage are essential for remyelination as they clear debris [20] and produce cytokines potentially useful for remyelination [31, 52]. Treg have now been identified in some MS lesions [13], and increasing Treg number in models of EAE aids recovery from disease [44]. Therefore, Sema3A pathway inhibition may lead to an increased number of NP1+ Treg in MS lesions and reduce effector T cell responses locally. However, we cannot test this in our mouse model as we do not see a T cell response.

NP1 is a receptor for vascular endothelial growth factor (VEGF) as well as Sema3A, although these ligands occupy distinct binding sites which can be targeted separately by blocking antibodies [26, 33] and binding stimulates association with different co-receptors (plexins for Sema3A and VEGFR1/2 for VEGF), and different downstream signalling pathways at least in neuronal cells (reviewed in [57]). The effect of VEGF signalling through the NP1 receptor on oligodendrocytes or on remyelination is not known, but NP1-null mice are embryonic lethal due to abnormalities of the development of the vascular system [19] and transgenic mice with mutation of the Sema3A binding site of NP1 only are viable [16], and so selective inhibition of the Sema3A binding site seems the preferred option. Sema3A-NP1 binding has been shown to be tractable, at least with a large molecule inhibitor obtained from fungus, which has been used to improve peripheral nerve regeneration in corneal transplants in mice [32], and axonal regeneration after spinal cord transection in rats [18].

This work shows the importance of OPC migration in the remyelination failure, giving a new group of therapeutic targets for aiding remyelination in MS patients, with the specific target of the Sema3A/NP1 pathway.

## Electronic supplementary material

Below is the link to the electronic supplementary material.

**Suppl. Fig. 1** NP1 and NP2 are also expressed by astrocytes and microglia/macrophage in human MS lesions (**a**) Astrocytes (GFAP-labelled) and microglia/macrophages (CD68-labelled) in MS lesions express NP1 (chronic active lesion shown for GFAP, active lesion for CD68). **(b)** Astrocytes and microglia/macrophages in MS lesions express NP2 (chronic inactive lesion shown for GFAP, active lesion for CD68). Examples of double-positive cells are labelled with arrows. Pictures are flattened confocal stacks. *Scale bar* 10 μm (EPS 15941 kb)

**Suppl. Fig. 2** Few oligodendroglial cells are proliferating or undergoing apoptosis 2 weeks after injection of LPC in mouse corpus callosum (**a**) There are few actively proliferating cells in the lesion 2 weeks after lesion induction (8 days after treatment with Sema3A or Sema3F) and most of these cells are not OPCs (as labelled by nkx2.2). There is no difference between the treatment groups. Sema3A treated lesion is shown. (**b**) There are also few cells undergoing apoptosis, at 2 weeks after lesion induction (8 days after treatment with Sema3A or Sema3F). Again, few of these are either OPCs (nkx2.2 + , two double-positive cells marked with arrows) or mature oligodendrocytes (CC1 +). There is no difference between the treatment groups. Sema3F treated lesion is shown. *Scale bar* 10 μm (EPS 12373 kb)

**Suppl. Fig. 3** Myelin thickness compared to axon diameter for control, Sema3A and Sema3F treated groups (**a**) Graphs show the g-ratio plotted against the axon diameter for individual fibres to show the distribution of results. A trend line (exponential) is added to each graph, with the mathematical equation used to define this. (**b**) The trend lines from each graph are plotted together for each time point to visualize differences. The Sema3A treated group has myelinated axons with thinner myelin (higher g-ratio) than control and Sema3F-treated animals (EPS 6172 kb)

**Suppl. Fig. 4** Myelin thickness compared to axon diameter for control, and Sema3AKD groups (**a**) Graphs show the g-ratio plotted against the axon diameter for individual fibres to show the distribution of results. A trend line (exponential) is added to each graph, with the mathematical equation used to define this. (**b**) The trend lines from each graph are plotted together for each time point to visualize differences. There is no difference between the trend lines between groups (EPS 2712 kb)
